# Integrative network analysis of nineteen brain regions identifies molecular signatures and networks underlying selective regional vulnerability to Alzheimer’s disease

**DOI:** 10.1186/s13073-016-0355-3

**Published:** 2016-11-01

**Authors:** Minghui Wang, Panos Roussos, Andrew McKenzie, Xianxiao Zhou, Yuji Kajiwara, Kristen J. Brennand, Gabriele C. De Luca, John F. Crary, Patrizia Casaccia, Joseph D. Buxbaum, Michelle Ehrlich, Sam Gandy, Alison Goate, Pavel Katsel, Eric Schadt, Vahram Haroutunian, Bin Zhang

**Affiliations:** 1 Department of Genetics and Genomic Sciences, Icahn School of Medicine at Mount Sinai, One Gustave L. Levy Place, 1470 Madison Avenue, New York, NY 10029 USA; 2 Icahn Institute of Genomics and Multiscale Biology, Icahn School of Medicine at Mount Sinai, One Gustave L. Levy Place, New York, NY 10029 USA; 3 Division of Psychiatric Genomics, Department of Psychiatry, Icahn School of Medicine at Mount Sinai, One Gustave L. Levy Place, New York, NY 10029 USA; 4 Friedman Brain Institute, Icahn School of Medicine at Mount Sinai, One Gustave L. Levy Place, New York, NY 10029 USA; 5 Psychiatry, JJ Peters VA Medical Center, 130 West Kingsbridge Road, Bronx, NY 10468 USA; 6 Seaver Autism Center for Research and Treatment, Icahn School of Medicine at Mount Sinai, One Gustave L. Levy Place, New York, NY 10029 USA; 7 Nuffield Department of Clinical Neurosciences, University of Oxford, Oxford, OX3 9DU UK; 8 Fishberg Department of Neuroscience, Icahn School of Medicine at Mount Sinai, One Gustave L. Levy Place, New York, NY 10029 USA; 9 Department of Pathology, Icahn School of Medicine at Mount Sinai, One Gustave L. Levy Place, New York, NY 10029 USA; 10 Department of Pediatrics, Icahn School of Medicine at Mount Sinai, One Gustave L Levy Place, New York, NY 10029 USA; 11 Departments of Neurology, Icahn School of Medicine at Mount Sinai, One Gustave L Levy Place, New York, NY 10029 USA; 12 The Alzheimer’s Disease Research Center, Icahn School of Medicine at Mount Sinai, One Gustave L Levy Place, New York, NY 10029 USA; 13 Ronald M. Loeb Center for Alzheimer’s Disease, Icahn School of Medicine at Mount Sinai, One Gustave L Levy Place, New York, NY 10029 USA

**Keywords:** Alzheimer’s disease, Dementia, Differential expression, Gene co-expression network, Gene module, Systems biology, Selective vulnerability, Demyelination, Brain cell types

## Abstract

**Background:**

Alzheimer’s disease (AD) is the most common form of dementia, characterized by progressive cognitive impairment and neurodegeneration. However, despite extensive clinical and genomic studies, the molecular basis of AD development and progression remains elusive.

**Methods:**

To elucidate molecular systems associated with AD, we developed a large scale gene expression dataset from 1053 postmortem brain samples across 19 cortical regions of 125 individuals with a severity spectrum of dementia and neuropathology of AD. We excluded brain specimens that evidenced neuropathology other than that characteristic of AD. For the first time, we performed a pan-cortical brain region genomic analysis, characterizing the gene expression changes associated with a measure of dementia severity and multiple measures of the severity of neuropathological lesions associated with AD (neuritic plaques and neurofibrillary tangles) and constructing region-specific co-expression networks. We rank-ordered 44,692 gene probesets, 1558 co-expressed gene modules and 19 brain regions based upon their association with the disease traits.

**Results:**

The neurobiological pathways identified through these analyses included actin cytoskeleton, axon guidance, and nervous system development. Using public human brain single-cell RNA-sequencing data, we computed brain cell type-specific marker genes for human and determined that many of the abnormally expressed gene signatures and network modules were specific to oligodendrocytes, astrocytes, and neurons. Analysis based on disease severity suggested that: many of the gene expression changes, including those of oligodendrocytes, occurred early in the progression of disease, making them potential translational/treatment development targets and unlikely to be mere bystander result of degeneration; several modules were closely linked to cognitive compromise with lesser association with traditional measures of neuropathology. The brain regional analyses identified temporal lobe gyri as sites associated with the greatest and earliest gene expression abnormalities.

**Conclusions:**

This transcriptomic network analysis of 19 brain regions provides a comprehensive assessment of the critical molecular pathways associated with AD pathology and offers new insights into molecular mechanisms underlying selective regional vulnerability to AD at different stages of the progression of cognitive compromise and development of the canonical neuropathological lesions of AD.

**Electronic supplementary material:**

The online version of this article (doi:10.1186/s13073-016-0355-3) contains supplementary material, which is available to authorized users.

## Background

Alzheimer’s disease (AD) is a complex neurodegenerative disease characterized by accumulation of amyloid plaques and neurofibrillary tangles (NFT) in the brain [1–3]. The development of AD causes irreversible and progressive loss of neurons resulting in cognitive impairment and ultimately dementia [4, 5]. As the disease progresses, more and more areas of the brain become damaged but AD does not affect all brain regions simultaneously or uniformly [6]. Some brain regions are more vulnerable to AD than others [7, 8]. Yet, the molecular basis of AD development and progression remains elusive.

Whole transcriptome analyses have shown much promise in understanding how altered gene expression contributes to complex diseases such as cancer [9], obesity [10], schizophrenia [11], and neurodegenerative disorders [12, 13]. The unbiased quantification and bioinformatic analysis of genome-wide RNA expression provides insights into biological pathways that regulate cellular processes and disease progressions at the molecular level. Transcriptome analysis has been widely applied to investigate the pathogenesis of AD in mouse models [14, 15] and human postmortem brain tissues [16–18]. Analysis of gene expression abnormalities in the human postmortem brain affected to greater or lesser degrees can identify genes and pathways dysregulated by AD. However, the power of transcriptomic analysis is hindered by the analysis of very limited number of brain regions and restricted severity stages in the previous studies of AD.

Although complex human diseases such as AD likely result from the interplay of many genetic and environmental factors involving thousands or tens of thousands of transcripts and proteins, core features of the disease can be characterized by studying affected molecular networks through the cognitive and neuropathological progression of the disease [19]. Gene co-expression network analysis approaches have been developed to capture interactions among genes and to identify higher order network structures such as modules comprising highly interconnected genes. Using a dataset consisting of gene expression profiles from laser-captured neurons from the middle temporal gyrus, entorhinal cortex, hippocampus, and posterior cingulate cortex from 34 AD patients and 13 controls [20], Ray and Zhang [21] constructed co-expression networks and found differential connectivity between region-specific networks enriched for two broad categories of functional pathways: inflammation/immune-related pathways and cytoskeleton remodeling pathways. More recently, Zhang et al. [18] performed a multiscale gene network analysis (MNA) of a much larger cohort of human brain specimens from dorsolateral prefrontal cortex (PFC), visual cortex (VC), and cerebellum (CB) in 376 AD patients and 173 non-demented controls. MNA revealed many facets of the molecular-interaction structures in AD and formally rank-ordered gene subnetworks based on their relevance to AD pathological and clinical traits. Using less sophisticated analytical approaches, Haroutunian et al. [16] staged regional transcriptional dysregulation based on the severity of global cognitive compromise.

In this study, we significantly expand the characterization of molecular networks associated with AD across multiple brain regions by generating and then analyzing a large-scale transcriptomic dataset [16] from 1053 postmortem brain tissues spanning 19 brain regions from 125 subjects with a full spectrum of AD severity in brains devoid of AD-unrelated confounding neuropathologies, such as cerebrovascular disease. Gene co-expression network analysis was applied to these data to identify subnetworks that were dysregulated in AD and/or associated with AD pathology. We further rank-ordered these subnetworks by the degree of dysregulation and association to AD to discover novel pathways and key genes that may serve as effective targets for therapeutic intervention.

## Methods

### Microarray gene expression profile and data preprocessing

The RNA samples collected from the current Mount Sinai Medical Center Brain Bank (MSBB) AD cohort were profiled on two Affymetrix microarray platforms, Human Genome (HG) U133A and U133B, except in two brain regions, amygdala (AMYG) and nucleus accumbens (NAc), for which the Affymetrix HG U133 Plus 2.0 array was used. Since there were a limited number of common probesets between HG U133A and HG U133B, the probesets from the two platforms were merged in the analyses, with signals of common probesets averaged. Affymetrix HG U133 Plus 2.0 includes all probesets on U133A and U133B and 9921 additional probesets representing approximately 6500 additional genes. The array probes were annotated according to the Ensembl version 72 (genome build GRCh37.p11; June 2013) using the R/Biomart library. The raw microarray data were first quantile normalized with all probesets on the arrays by making use of the RMA [22] method implemented in the R/Bioconductor package affy (v1.44) with the default parameters and then corrected for covariates including sex, postmortem interval (PMI), pH, and race using a linear regression model.

### An integrative network approach to analyze the MSBB data

**Fig. 1:**
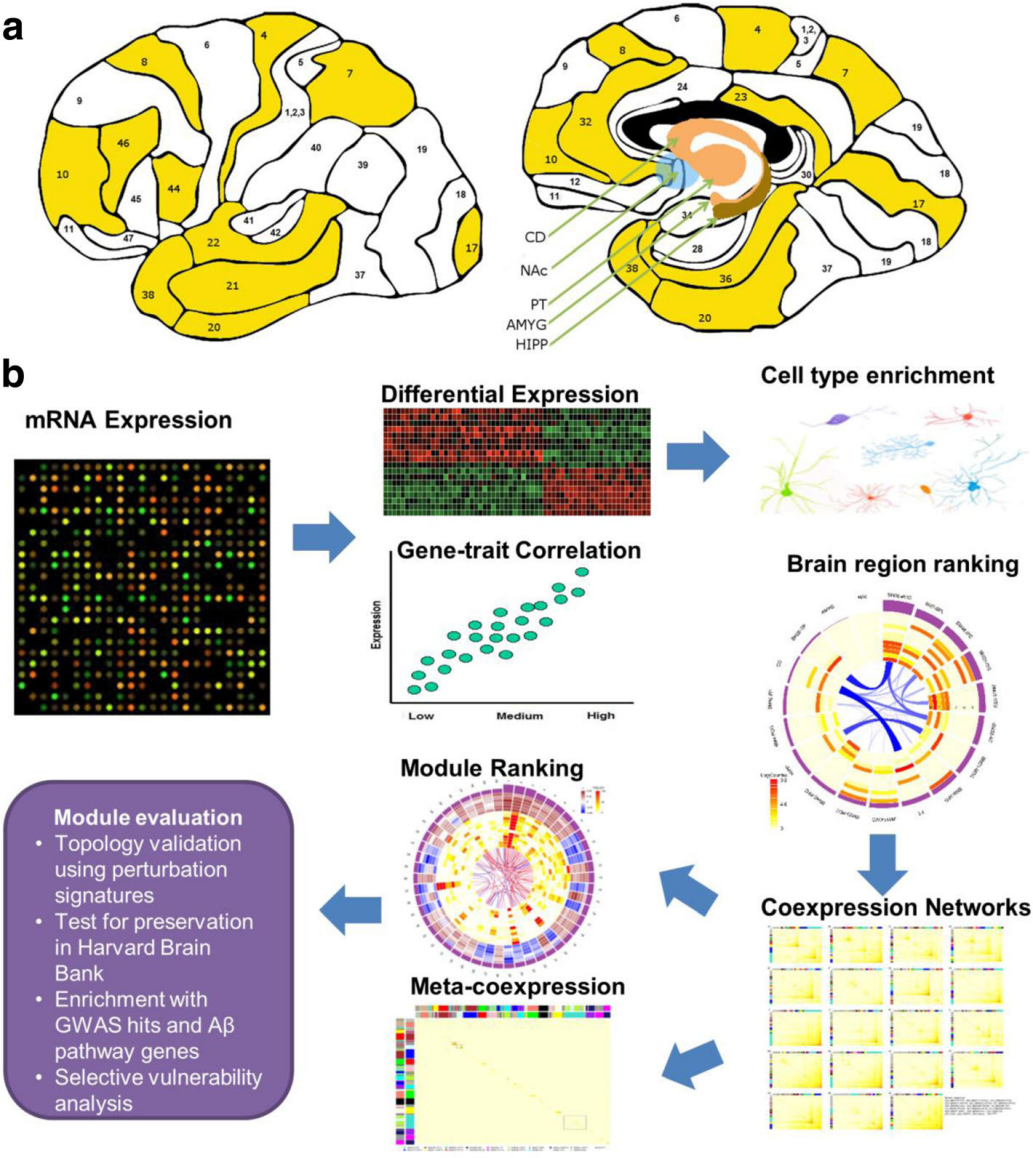
Data generation and analysis flow. **a** *Schematic illustration* of the 19 brain regions profiled in the current study. The *numbered areas* highlighted in *yellow* are the Brodmann (BM) areas, while the *arrows* indicate caudate nucleus (CD), nucleus accumbens (NAc), putamen (PT), amygdala (AMYG), and hippocampus (HIPP), respectively. **b** An overview of the analysis flow. RNA samples from 19 brain regions of 125 MSBB specimens were collected and profiled using Affymetrix Genechip microarrays. From the microarray RNA expression data, we first identified gene signatures associated with cognitive/neuropathological outcomes through differential expression and gene-trait correlation analyses. We tested enrichment of cell type-specific genes in the differentially expressed gene signatures and rank-ordered brain regions in relevance to AD by comprehensively comparing the number of gene signatures identified in each region for each trait. Next, we constructed a gene co-expression network for each brain region. Based on the network modules identified in individual brain regions, we constructed a meta-co-expression network to assess the correlation of networks between brain regions. Then we rank-ordered the co-expression network modules across all brain regions by multiple features. We evaluated the network module topology using gene perturbation signatures. Then, for top modules, we tested the replication of the network modules in an independent dataset from the Harvard brain bank. Later, we examined the cell type specificity and enrichment of genetic signal of the top ranked modules by using AD susceptibility genes and Aβ pathway genes. Finally, we explored regional selective vulnerability to the disease with two example pathways

We applied an integrative network-based approach to identify critical genes and gene networks associated with AD in 19 brain regions (Fig. 1a and b). We first identified gene signatures associated with clinical/neuropathological outcomes through differential expression (DE) and gene-trait correlation analyses. We tested enrichment of cell type-specific genes in the DE signatures and rank-ordered brain regions in relevance to AD by the number of gene signatures associated with different clinical/neuropathological traits. Next, we computed gene-gene correlations and performed hierarchical clustering analysis to construct co-expression networks for each brain region. Based on the network modules identified in individual brain regions, we constructed a meta-co-expression network to assess the correlation of networks between brain regions. Then we rank-ordered the co-expression network modules across all brain regions by multiple features. We evaluated the network module topology using gene perturbation signatures. Then, for top modules, we tested the replication of the network modules in an independent dataset from the Harvard brain bank. Later, we examined the cell type specificity and enrichment of genetic signal of the top ranked modules by using AD susceptibility genes and Aβ pathway genes. Finally, we explored regional selective vulnerability to the disease with two example pathways.

### Differential expression analysis

We first computed the correlations between gene expression and six neuropathological or cognitive traits, including clinical dementia rating (CDR), Braak stage, Consortium to Establish a Registry for Alzheimer’s Disease (CERAD) diagnostic certainty, plaque density mean, sum of neuritic plaque (NP) density estimates, and sum of NFT density estimates. For each trait, the samples were classified into three groups according to the disease status and severity staging defined by the trait: normal, low severity, and high severity. Additional file 1: Table S1 tabulates the complete sample demographic information and Additional file 1: Table S2 shows the criteria for defining these disease staging groups with respect to each trait. We applied a linear model analysis to identify genes differentially expressed among the disease staging groups by using R package Limma (v3.26.9) with default parameters [23]. To adjust for multiple tests, false discovery rate (FDR) was estimated by fitting the same Limma linear models after repeatedly reshuffling sample group labels (five times) to derive an empirical null distribution of the test statistics (limma moderated t-statistics), and then FDR at a cutoff was estimated as *FDR* = *n* × (*FP*/*N*)/*P*, where *P* denotes the number of significant tests at a given cutoff in the non-permuted data, *n* is the number of tests in the non-permuted data, *FP* is the number of false positives at a given cutoff from the permutation, and *N* is the total number of tests in permutation. This procedure is essentially the same as first computing empirical *P* values based on a null distribution from permutation and then applied Benjamini–Hochberg’s (BH) FDR control [24] with the empirical *P* values. Probesets with a FDR less than 0.05 and fold change (FC) larger than 1.5 were considered significant.

### Correlations between gene expression and cognitive/neuropathological traits

Complementing the differential expression analysis defined above, we carried out correlation analyses to identify gene expression traits that were positively or negatively correlated with each of the six cognitive/neuropathological traits described above. Since CDR, Braak, and CERAD were measured as discrete ordinal scores, Spearman’s rank correlation coefficient analysis was used to compute the strength of correlation between these clinical/neuropathological traits and gene expression traits. FDR was estimated by first computing a null distribution of Spearman’s correlation coefficients through permutation of trait phenotypes (five times) and then applying a similar FDR control procedure as described in differential expression analysis. We used a FDR of 0.05 as the significance threshold.

### Accessing brain cell type-specific gene signatures

To characterize if certain brain cell types were dysregulated in disease, we computed a panel of cell type-specific genes for five major brain cell types, including astrocytes, endothelial, neurons, microglia, and oligodendrocytes by making use of a large scale human brain single-cell RNA-sequencing (RNA-seq) dataset [25]. We downloaded RNA-seq read count data from GEO (accession no. GSE67835) and selected samples corresponding to the five major brain cell types: astrocyte, endothelial, microglia, neuron, and oligodendrocyte. Genes with less than 50 reads across all samples were discarded. The remaining gene count data were analyzed by a Bayesian negative binomial regression model with cell type identity, basal expression (or library size), and subject source incorporated as predictors by making use of the RStan source code provided in [26]. Using numerical samples obtained by Markov chain Monte Carlo (MCMC), we calculated the posterior probability that gene expression was enriched in one cell type compared to basal expression given by the regression. A gene was considered cell type-specific if it met two criteria: (1) it was enriched with 99.9 % posterior probability in one cell and not enriched in any other cells; and (2) its expression in the enriched cell was on average fivefold larger than basal expression in the numerical samples. The inferred brain cell type-specific gene signatures are provided in Additional file 1: Table S3.

### Set enrichment analysis

Set enrichment analysis (or set overlap test) was carried out using Fisher’s exact test assuming the sets of genes, such as differentially expressed genes (DEGs), module genes, and network neighbors, were identically independently sampled from the genome-wide genes profiled by the array. To control for multiple testing, we employed the BH approach to constrain the FDR. For functional enrichment analysis of signature genes, the gene ontology (GO) annotations and canonical pathways (Biocarta, KEGG and Reactome) gene sets were obtained from the Molecular Signatures Database (MSigDB) v4.0 [27].

### Co-expression network analysis

Weighted gene co-expression network analysis (WGCNA) [28] was performed to identify the gene modules with coordinated expression patterns for each brain region. Briefly, Pearson’s correlation coefficients were calculated between all pairs of probesets after microarray data normalization. Next, the correlation matrix was converted into an adjacency matrix using a power function *f*(*x*) = *x* ^*β*^, where *x* was the element of the correlation matrix and parameter *β* was determined such that the resulting adjacency matrix was approximately scale-free [28]. In the present study, we used *β* = 6 with other parameters set by default and this led to a truncated scale-free index greater than 0.95 for all the 19 co-expression networks. The adjacency matrix was subsequently transformed into a topological overlap matrix (TOM) [29] which captured both the direct and indirect interactions between a pair of probesets. Average linkage hierarchical clustering was then employed to cluster probesets based on the TOM. Finally, a tree cutting algorithm [30] was used to dynamically cut the hierarchical clustering dendrogram branches into highly connected modules, each of which was assigned a distinct color code. The whole network construction procedure was based on an R package WINA, a computationally optimized version of the WGCNA package.

### Sort brain regions and network modules using an ensemble ranking metric

For each clinical/neuropathological trait, we had performed differential expression between any pair of disease severity groups and also called trait associated genes (TCGs). The number of DEGs (or TCGs) identified from different brain regions could be regarded as a variable (or feature) for ranking order the brain regions in relevance to the variation of a particular trait. In total, there were 24 variables useful for ranking: six sets of TCGs, and 18 sets of DEGs including three sets (i.e. medium versus low, high versus medium, and high versus low) from each of the six traits. To congregate rankings from all 24 variables, we proposed to compute a composite importance score of a brain region *i* as the geometric mean of the functions of all ranking variables:

$$ {S}_i={\left({\prod}_{j\kern0.5em =\kern0.5em 1}^nf\left({K}_{ij}\right)\right)}^{1/n} $$

where *n* = 24 denotes the number of ranking variables, *K*_*ij*_ denotes the number of genes identified for *i*th brain region regarding *j*th ranking variable, and *f* is a transformation function. Here we used log transformation function as it can shrink the gene counts of different variables to a more comparable scale. The choice of *f* is beyond the scope the present study. Then the composite importance scores were scaled to be in the range of 0–1 by dividing the maximum score. Finally, the brain region with the highest composite importance score was ranked in the top, while the brain region with the lowest composite importance score was ranked in the bottom.

We applied the above ensemble ranking metric to rank-order co-expression network modules. For each module, the ranking variables included the *P* values of strength of correlations between module eigengene expression and clinical/neuropathological traits and *P* values of enrichment for DEGs and TCGs. We used minus log as the transformation function for *P* values.

## Results

### Development of a large AD cohort

A total of 125 human brains were accessed from the Mount Sinai/JJ Peters VA MSBB cohort, which holds over 1800 well-characterized brains. This cohort was assembled after applying stringent inclusion/exclusion criteria and represents the full spectrum of clinical and neuropathological disease severity in the absence of discernable non-AD neuropathology. All neuropsychological, diagnostic and autopsy protocols were approved by the Mount Sinai and JJ Peters VA Medical Center Institutional Review Boards. Neuropathological assessments, cognitive, and medical and neurological status determinations were performed according to previously published procedures as described in detail [16]. For each sample, a number of cognitive and neuropathological outcomes were recorded and analyzed herein, including CDR, Braak (Braak NFT score) [31, 32], CERAD diagnoses and ratings of pathology (Consortium to Establish a Registry for Alzheimer’s Disease diagnosis) [33], plaque density mean (PLQ_Mn, average of NP counts in five cardinal cortical regions), sum of neuritic plaque density estimates (NPrSum, sum of CERAD semi-quantitative rating scores for all cortical regions examined neuropathologically), and sum of neurofibrillary tangles density estimates (NTrSum, sum of semi-quantitative NFT density ratings for all cortical regions examined). Detailed sample demographic information is provided in Additional file 1: Table S1 and a brief description of the cognitive and neuropathological traits is provided in Additional file 1: Table S2a.

RNA samples from 19 brain regions (Fig. 1a) isolated from the 125 MSBB specimens were collected and profiled using Affymetrix Genechip microarrays as described in “Methods” (Additional file 1: Table S2b). RNA quality was assessed using a combination of a 260/280 ratio derived from a high resolution electrophoresis system (LabChipTM, Agilent Technologies, Palo Alto, CA, USA) and 3’–5’ hybridization ratios for glyceraldehyde-3-phosphate (GAPDH) probes. Not all brain regions for all subjects were available for analysis. There was an average of 55 subjects per brain region with varying degrees of AD pathological and cognitive abnormalities. After data preprocessing, we used an integrative network approach to identify critical genes and gene networks associated with AD (see “Methods” and Fig. 1b for details).

### Correlation analysis of cognitive and neuropathological traits

**Fig. 2:**
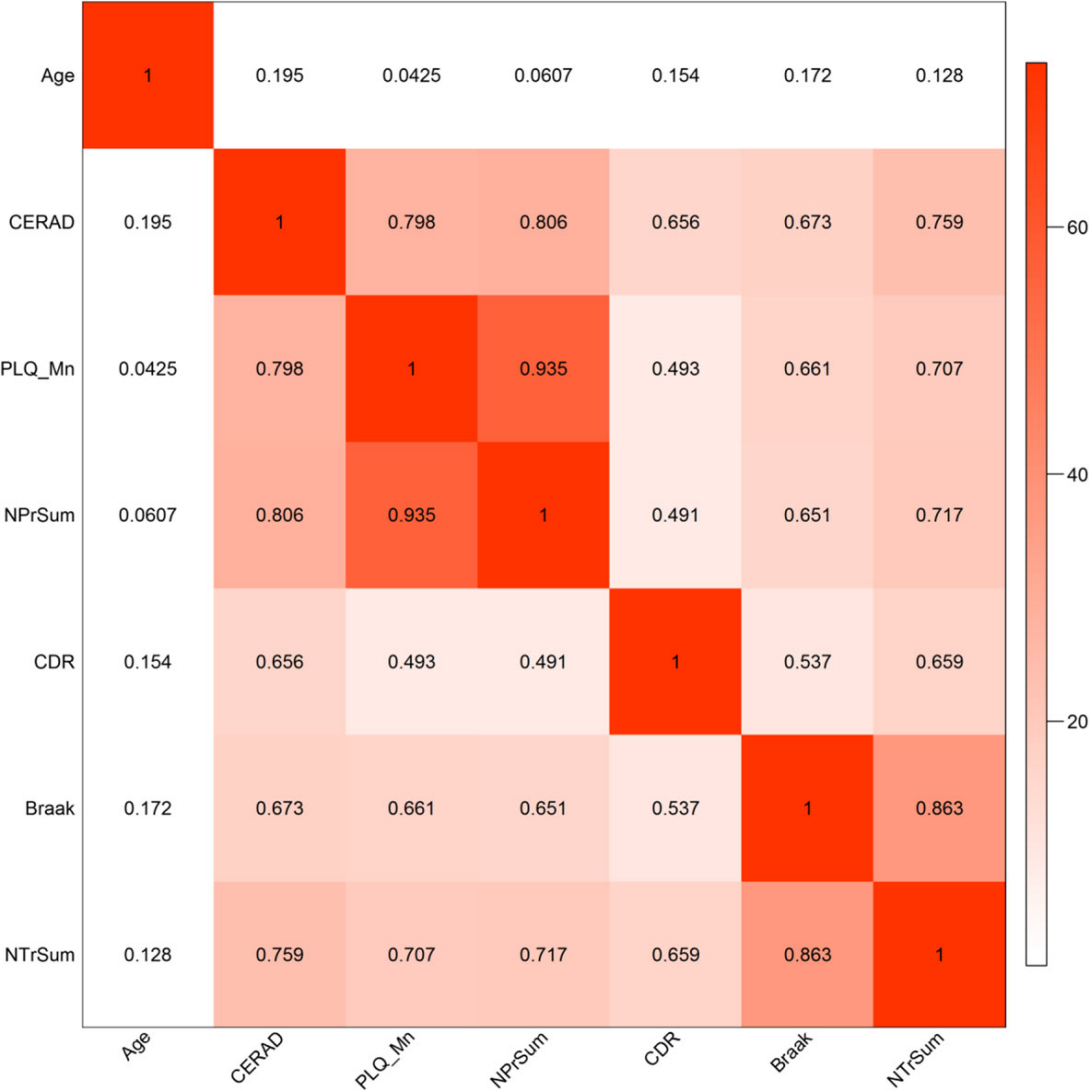
Correlations among age, cognitive, and neuropathological traits. The *number* in each cell indicates the Spearman’s correlation coefficient between row and column variables, with color intensity indicating the *P* value at minus log 10 scale

Figure 2 shows the Spearman correlation coefficients among age and the cognitive and neuropathological traits analyzed across all samples. All neuropathological traits were highly positively correlated with the cognitive status outcome CDR, which is consistent with both NFTs and NP being strongly associated with cognitive decline in AD [3]. Age was not correlated with any of the cognitive and neuropathological traits at a threshold of 0.01 for correlation *P* value. Though it is known that the gene expression and some indices of neuropathology might be related to age, we chose not to correct for age for two reasons. First, age is a risk factor to AD and correction for age would lose/weaken disease signal. Second, more than 81.4 % of the genes differentially expressed regarding disease traits in age un-corrected data were also detected from age corrected data (data not shown), suggesting that the impact of age on the analysis is very small in this study.

### Differential expression analysis

For each cognitive or neuropathological trait, we separated samples into three groups, normal, low disease severity, and high disease severity (Additional file 1: Table S2c) and then performed differential gene expression between any two groups for every brain region using a linear model analysis. We followed previous practices to subdivide samples regarding Braak tangle staging (0–2, 3–4, and 5–6) [34], CDR (0, 0.05–2, 3–5) [16], and CERAD (normal, possible or probable AD, definite AD) [16, 35]. To the best of our knowledge, we are the first to systematically analyze gene expression changes associated with the three neuropathological quantitative traits, plaque density mean, sum of NP density estimates across multiple cortical regions, and sum of NFT density estimates across multiple cortical regions. For each of these three traits, we assigned clean brains (without plaque or tangle) as normal and then divided the remaining brains into low and high severity groups with roughly equal numbers of sample size. At a FDR of < 0.05 and FC > 1.5, we detected a total of 6037 probesets across 19 brain regions and six traits (Additional file 1: Table S4a). Additional file 1: Figure S1a illustrates the number of DEGs. The number of DEGs varied greatly between the different brain regions and traits. For example, DEGs were detected in 11 brain regions when stratification was based on cognitive status (CDR) while DEGs were detected in only two brain regions for sum of NFT density estimates. Of course, the absolute numbers of brain regions associated with any given trait varied depending on the FDR and FC thresholds set.

A number of 34 AD risk genes have been identified so far, including *APOE*, *APP*, *BIN1*, *PSEN1*, *PSEN2*, and *TREM2* (reviewed in [36]). We studied whether these genes were dysregulated in low and/or high severity status as defined by each of the six cognitive/neuropathological traits. As shown in Additional file 1: Table S4b, different brain regions showed different patterns of gene expression dysregulation emerged for *PSEN1*, *MEF2C*, *PICALM*, and *PLD3* depending on the cognitive or neuropathological trait under investigation. The brain regions significantly associated with altered expression of the transcripts of these genes included the inferior temporal gyrus (BM20), the middle temporal gyrus (BM21), and the inferior and superior frontal gyri (BM44 and BM8).

**Fig. 3:**
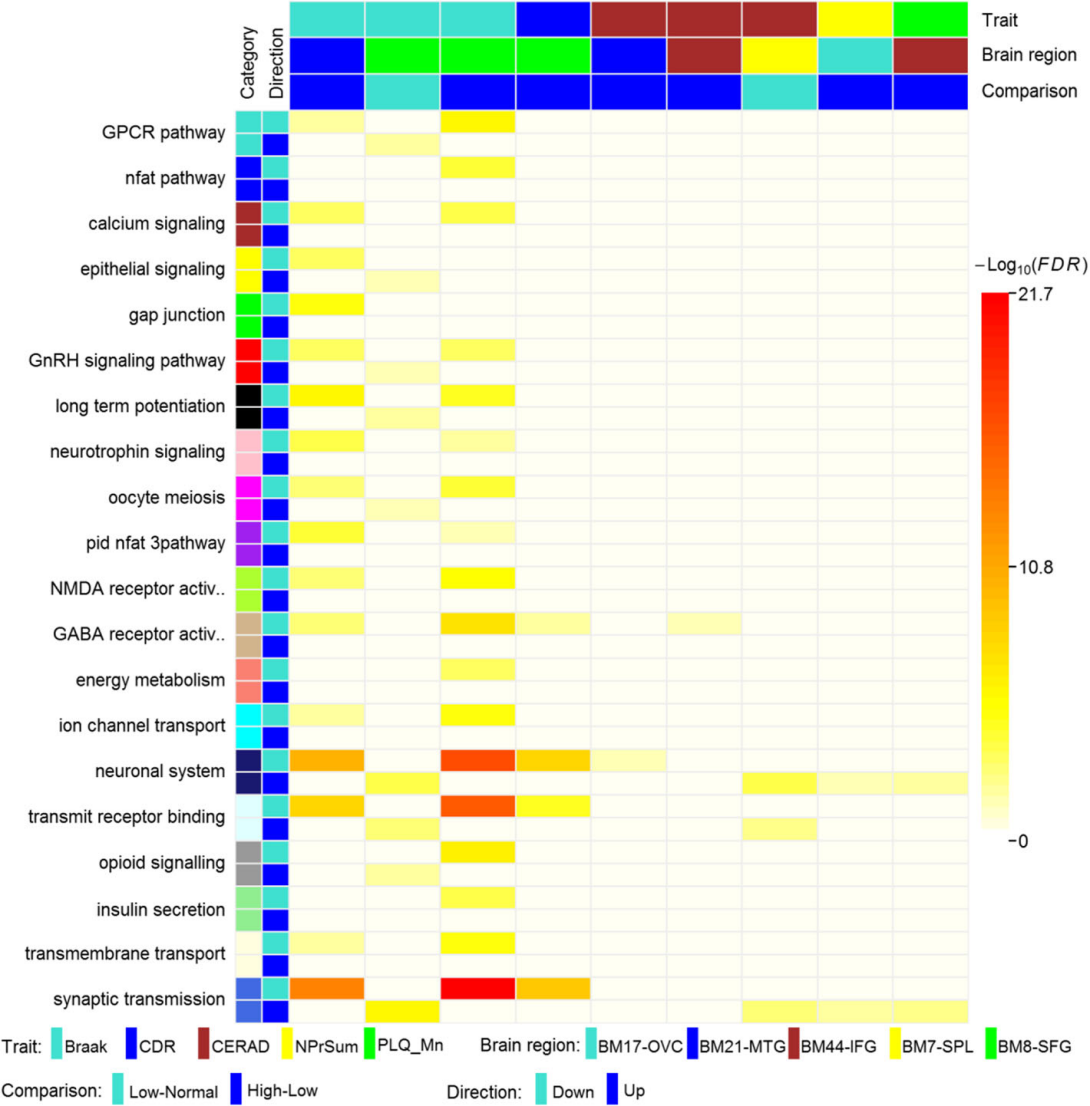
*Heat map* showing the top functional pathways enriched in the DEGs identified between low and normal severity groups and between high and low severity groups with respect to each of the six cognitive/neuropathological traits. The *heat map* color intensity denotes the statistical significance of the enrichment as calculated from Fisher’s exact test after correction for multiple tests

We next tested whether specific GO and functional pathway terms were enriched within the DEG signatures using the MSigDB gene annotation database (Fig. 3 and Additional file 1: Tables S5a and b). The DEGs, especially the downregulated genes, between the high and low Braak neuropathology stage in the superior frontal gyrus (BM8) and the middle temporal gyrus (BM21), presented the most significant enrichment of signaling pathways such as GPCR pathway, calcium signaling, neurotrophin signaling, opioid signaling, epithelial signaling, and GnRH signaling. As expected, several well-established pathways such as GABA A receptor activation, neuronal systems, neurotransmitter receptor binding, and synaptic transmission were associated with some disease severity traits in multiple brain regions. However, these pathways may change in different directions (i.e. upregulation and downregulation) in different brain regions. For example, the synaptic transmission pathway was enriched for the downregulated genes between high and low Braak stages and between severe and minor dementia, but this same pathway was enriched for upregulated genes during early stages of disease, i.e. low Braak stage versus controls or low CERAD versus normal brain in the superior frontal gyrus (SFG) or the superior parietal lobule (SPL). Although additional molecular studies in postmortem human brain and animal studies are needed to confirm these complex relationships, the results described here are consistent with a hypothesis of compensatory upregulation of genes in this pathway in early disease states followed by their significant downregulation as the disease progresses.

#### Neuron-specific, oligodendrocyte-specific, and astrocyte-specific genes were most enriched in the DEG signatures

**Fig. 4:**
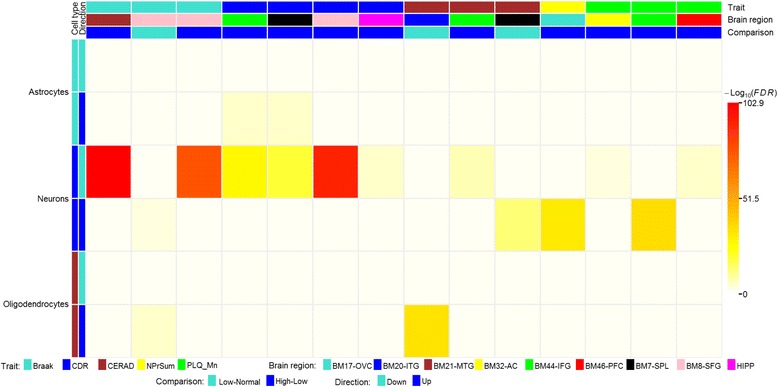
Cell type specificity of the DEG signatures identified between low and normal severity groups and between high and low severity groups for each of the six cognitive/neuropathological traits. The *heat map* color intensity denotes the statistical significance of the enrichment as calculated from Fisher’s exact test after correction for multiple tests

In complex neurodegenerative diseases such as AD, there is mounting evidence that the different cell types that comprise the human brain are targeted differentially and may be affected at different stages of the disease. To interrogate if particular cell types were more or less susceptible to dysregulation, we compiled a number of gene signatures specific for astrocytes, endothelial cells, microglia, neurons, and oligodendrocytes (see “Methods”). As expected, using Fisher’s exact test, we found the gene signatures specific to neurons were most significantly enriched in the DEGs identified in multiple regions with respect to multiple traits (Additional file 1: Table S6 and Fig. 4). The neuron specific genes were primarily enriched for downregulated gene signatures of high versus low with respect to the traits Braak in regions BM8-SFG and BM21-MTG, CDR in regions BM7-SPL, BM8-SFG, BM44-IFG, and HIPP, CERAD in region BM44-IFG, and plaque density mean in regions BM32-AC and BM46-PFC. The neuronal specific genes were also found to be enriched for upregulated genes of high versus low when comparing plaque density mean in BM44-IFG and sum of NP density estimates in BM17-OVC, low versus normal for trait CERAD in BM7-SPL and Braak in BM8-SFG. Not surprisingly, astrocyte-specific genes followed a similar pattern of enrichment for upregulated genes in high versus low comparisons with respect to CDR in BM44-IFG and BM7-SPL.

It is of interest that oligodendrocyte specific genes, such as *UGT8*, which encodes a key enzyme involved in lipid biosynthesis in myelinating oligodendrocytes, were also enriched for DEGs as a function of brain region and disease severity. For example, the oligodendrocyte-specific genes were significantly enriched with upregulated DEG signatures when comparing CERAD or Braak stage low to normal in the ITG (BM20) and the SFG (BM8), respectively. These observations indicate that oligodendroglials become involved relatively early during the disease process, when neuron-specific DEGs are also upregulated (see above), and that their involvement may not necessarily be a natural consequence of axonal degeneration.

### Gene-trait correlation analysis

Complementing the differential expression signatures comprising expression traits that vary between severity groups defined by each trait, we also identified TCGs whose expression levels were positively or negatively correlated with the cognitive and neuropathological variables through Spearman’s rank correlation analysis. TCG analysis aimed to identify genes showing trend-like expression response to disease progress that may be otherwise missed by differential expression analysis. Additional file 1: Table S7 lists the 1215 TCGs identified at a FDR threshold of 0.05 for each of the six traits. As illustrated in Additional file 2: Figure S1b, the number of TCGs varied dramatically across brain regions and traits. More than 84 % of the TCGs were identified from three brain regions for three different traits, including 759 TCGs from the putamen (PT) associated with CDR, 150 TCGs from the region of parahippocampal gyrus (BM36-PHG) associated with mean cortical neurotitc plaque density (PLQ-Mn), and 118 TCGs from the superior temporal gyrus (BM22-STG) associated with the sum of cortical neurofibrillary tangle density ratings (NTrSum). The most significant TCG is *METTL13* (methyltransferase like 13), which was correlated with sum of NFT density estimates in the superior parietal lobule (BM7-SPL) (*r* = 0.70, *P* value = 1.25 × 10^–8^). The protein product encoded by this gene is the antiapoptotic protein FEAT, which is also aberrantly overexpressed in various human cancer tissues [37]. Among the list of the top trait correlated genes is *AKT2*, which encodes a serine/threonine-protein kinase Akt. The Akt kinase is a downstream mediator of the PI3K pathway and can phosphorylate a wide range of transcription factors and kinases such as GSK-3β. Akt regulates multiple biological processes including cell signaling, cell survival, proliferation, growth, and glycogen metabolism and the PI3K/AKT/GSK-3β has been shown to be implicated in multiple studies of AD including hyper-phosphorylation of Tau [38].

The GO categories and functional pathways significantly overrepresented in the TCGs are summarized in Additional file 1: Table S8. At a FDR threshold of 0.05, we only identified functional enrichment for the TCGs negatively correlated with dementia severity (CDR) in the PT. The most significant functional terms include several energy metabolism related pathways and cellular components, such as oxidative phosphorylation, TCA cycle and respiratory electron transport, and mitochondria. Increasing evidence implicates a role for mitochondrial dysfunction and oxidative damage in the pathogenesis of AD [39–41]. One possible mechanism of oxidative stress pathogenesis in AD is that abnormal mitochondria produced prominent neuronal oxidative stress in the surrounding cytoplasm which caused cytoplasmic damage in susceptible neurons [42]. Alternatively, it has been argued that the pathogenesis of AD is, at least in part, associated with reduced energy metabolism [43]. It is noteworthy that mitochondrial abnormalities have been linked to CDR in previous studies [44, 45]. Several neurodegenerative disease gene sets are also enriched in the TCGs negatively correlated with CDR in the PT, including the Parkinson’s disease KEGG pathway (fold enrichment (FE) = 5.6, FDR adjusted *P* value = 3.5 × 10^–4^), the Huntington’s disease KEGG pathway (FE = 6.3, FDR adjusted *P* value = 2.8 × 10^–3^), and the AD KEGG pathway (FE = 5.0, FDR adjusted *P* value = 0.011).

### Brain region interaction and ranking in relevance to AD

**Fig. 5:**
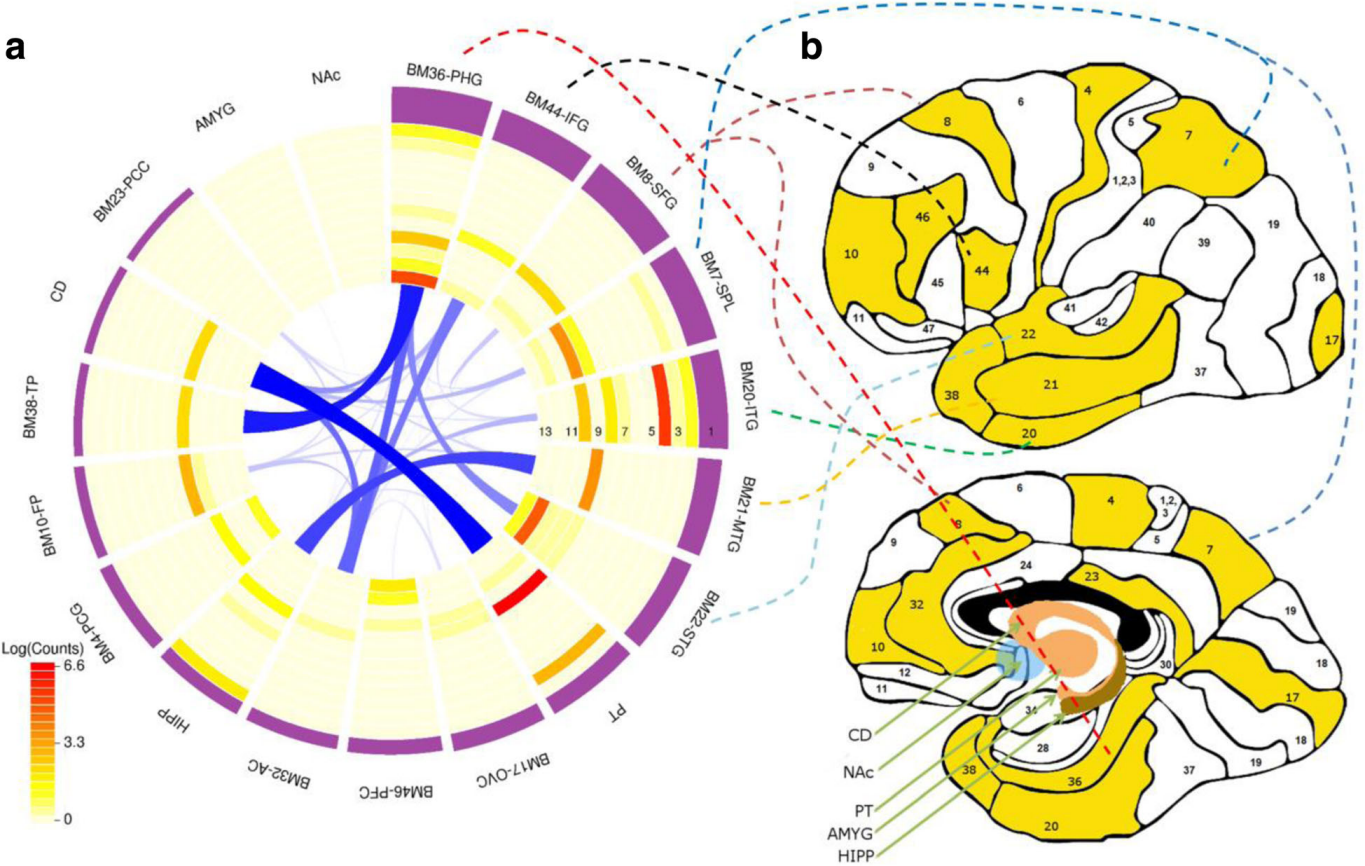
Brain regions rank-ordered by the relevance to AD pathology. **a** Brain regions rank-ordered by the number of DEGs and TCGs with respect to six cognitive/neuropathological traits. From outside to inside, the *bar chart* in the first track shows the scaled ranking scores with the bar height proportional to the ranking score, the *heat maps* in tracks 2–7 show the ranking of regions by the number of DEGs between high and normal severity groups with respect to the traits CDR, Braak, CERAD, plaque density mean, sum of NP density estimates, and sum of NFT density estimates, respectively; the *heat maps* in tracks 8–13 show the ranking of regions by the number of TCGs for traits CDR, Braak, CERAD, plaque density mean, sum of NP density estimates, and sum of NFT density estimates, respectively; while the size and color intensity of the *ribbons* in the *center* show the number of correlated gene pairs at FDR < 0.05 between any two brain regions. The legend color intensity shows the number of DEGs/TCGs with respect to a trait at log scale. **b** The locations of the top ranked brain regions in (**a**) are *highlighted* by a *dotted line*

We have computed the interactions among brain regions by the pairs of correlated microarray probesets between any two brain regions. As illustrated in Fig. 5a, strong interactions were detected between several brain regions with strong physical interconnectivity. For example, the PT and caudate nucleus (CD), two closely linked regions in the dorsal striatum that form the main components of the basal ganglia, presented more than 8.8 million significant probeset pairs which accounted for 0.4 % of the total number of probeset pairs at FDR of 0.05. Similarly, the parahippocampal gyrus (BM36-PHG) and the temporal pole (BM38-TP), two adjacent regions located in the temporal lobe, presented 7.9 million significantly correlated probeset pairs. Strong interactions were also found between the middle temporal gyrus (BM21-MTG) and the hippocampus (HIPP), between the anterior cingulate (BM32-AC) and the CD, and between the anterior cingulate gyrus and the parahippocampal gyrus.

Based on the differential gene expression and gene-trait correlation, we could rank-order brain regions in relevance to AD by comparing the number of DEGs and TCGs identified in each brain region with respect to cognitive or neuropathological trait. Using an ensemble ranking metric that congregates rankings from multiple sorting features as described in “Methods,” we ranked the 19 brain regions as shown in Fig. 5a. While sample sizes were different among the brain regions, which, as a result, might impact the power in detecting the DEGs and TCGs, we found this was not the case in the current dataset as ranking score and sample size were not correlated (Pearson’s correlation coefficient = 0.18, *P* = 0.458).

Several regions from the temporal lobe were ranked at the top, including the majority of the temporal cortical regions examined (Fig. 5a and b). The top ranked temporal cortex from this analysis is consistent with the roles of the temporal cortex in cognitive processes (e.g. perception of sensory input (visual, auditory, olfactory, and gustation), language comprehension, and memory formation and recall) as well as neuroimaging and neuropathological findings that identify the temporal lobe as the brain region closely associated with dementia onset and the earliest stages of AD and mild cognitive impairment [46, 47]. This study is perhaps the first effort to provide a comprehensive and objective ranking of many brain regions involved in AD based on unbiased molecular evidence and underscores the significance of several regions in temporal lobe to AD and its etiopathogenesis.

### Gene co-expression network analysis

AD, like many other phenotypes, is a complex process involving dysregulation of genes in different pathways. Since genes within the same pathway may show similar expression profiles, we employed weighted gene co-expression network analysis (WGCNA) to capture the coordinated gene expression for each brain region separately. The co-expression networks are illustrated by heat maps of topological overlap matrix (TOM) plots (Additional file 2: Figure S2). In each of the TOM plots, the rows and columns represent the same set of genes sorted by the hierarchical clustering tree of TOM with modules represented by colored labels. The number of modules identified from different brain regions ranged from 56 in the frontal pole (BM20-ITG) to 111 in the precentral gyrus (BM4-PCG). The fraction of genes that were successfully assigned into modules was quite similar: 79.3 ± 7 %.

The co-expression network modules were annotated with functional categories using gene set enrichment analysis. The top functional terms for each of the network modules are listed in Additional file 1: Table S9. Across all of the network modules, peptide chain elongation and ribosome genes were the most significantly enriched functional pathways. In fact, peptide chain elongation and ribosome were significantly enriched in at least one module in each of the 19 gene co-expression networks. For example, 65 of the 82 genes in module chocolate of brain region superior temporal gyrus (BM22-STG) were annotated with the peptide chain elongation pathway, resulting in more than 192-fold enrichment (FDR = 3.19 × 10^–147^). Peptide chain elongation is the process of linking together amino acids to extend the growing protein chain during protein biosynthesis in the ribosome. How these peptide chain elongation or ribosome enriched molecular processes influence the AD phenotype remains to be elucidated. Among the top functional categories were a number of immune response pathways, e.g. immune system and interferon signaling. Interestingly, we also found 37 modules enriched with the AD KEGG pathway, e.g. the yellow module of the network for the brain region PT presented a 3.5-fold enrichment of this pathway (FDR adjusted *P* value = 1.56 × 10^–19^).

The co-expression network analysis identifies gene modules, i.e. groups of genes, which show highly correlated expression profiles across samples. As a result, the pattern of correlated expression facilitates reduction of the module expression profile to one representative feature, the module eigengene [48], which is specifically defined as the first principal component of the standardized module expression data. It has been demonstrated that eigengenes among different modules often exhibit correlations, allowing for the construction of co-expression networks from eigengene expression profiles, similar to the construction of these networks using gene expression data [49]. To investigate how the network modules in different brain regions interact, we constructed a co-expression network based on the eigengene correlations among all the modules in each brain region gene co-expression network. For simplicity, the eigengene based network is referred to as the meta-co-expression network and the modules from the meta-co-expression network are referred to as meta-modules.

**Fig. 6:**
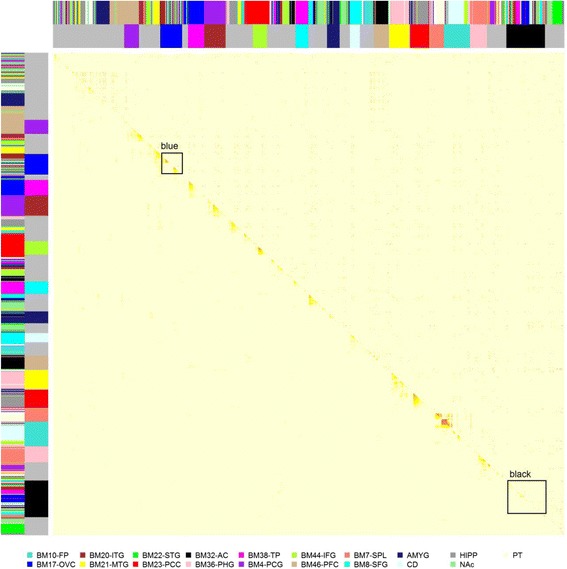
The meta-co-expression network constructed from module eigengenes identified across 19 brain regions. The *lower triangular* of the *heat map* shows the topological overlap matrix (TOM) while the *upper triangular* of the *heat map* shows the eigengene conservation across brain regions in terms of gene membership as measured by Jaccard index. The *outer color bars* along the *x- and y-axes* denote the origin of brain region for each eigengene and the *inner color bar* denotes the meta-module membership. Most of the meta-modules are brain region specific except the two *highlighted* in *rectangles*, i.e. *blue* and *black*

Fifteen meta-modules were identified from the meta-co-expression network analysis (Fig. 6 and Additional file 1: Table S10). Among the meta-modules, 13 were composed primarily of eigengenes from single brain regions (ranging from 70 % to 100 % of the meta-module members), reflecting tissue-specific correlation structures among 13 brain regions. The six brain regions not reflected in the brain region specific meta-modules are BM10-FP, BM20-ITG, BM21-MTG, BM22-STG, BM44-IFG, BM8-FC, and NAc. Eigengenes from these six brain regions participated in two different meta-modules: black and blue (highlighted in Fig. 6). The black meta-module consists of eigengenes from 17 brain regions, but with roughly 53 % of the eigengenes coming from four brain regions BM17-OVC (20.3 %), BM20-ITG (11.0 %), BM36-PHG (11.0 %), and BM38-TP (10.1 %). The blue meta-modules comprised eigengenes from 18 brain regions, with brain region BM20-ITG as the major contributor, accounting for more than 27.3 % of the eigengenes.

We further explored the conservation of modules across the brain regions by computing the similarity between all pairs of modules in terms of gene membership using the Jaccard index measure: $\frac{\left|A{\displaystyle \cap }B\right|}{\left|A{\displaystyle \cup }B\right|}$, i.e. the size of the overlap divided by the size of the union of the gene sets of two modules *A* and *B*. Of all pairs of modules tested, 1037 were found with a Jaccard index > 0.5, suggesting the presence of putative consensus modules in different brain regions. Interestingly, the members of the blue meta-module were enriched for immune response related functional pathways, including immune system, systemic lupus erythematosus, response to external stimulus, leishmania infection, allograft rejection, interferon signaling, IL6_7 pathway, and response to stress (Additional file 1: Table S10), suggesting that immune response is a common and coordinated feature of the multiple brain regions studied. Another immune-related gene present in co-expression modules of 19 brain regions examined was *TYROBP* the binding partner of which, *TREM2*, has recently been identified in strong association with AD. In the blue meta-module comprising eigengenes from 18 brain regions, *TYROBP* was present in 17 region-specific members. These observations are not only consistent with historical observations of immune/inflammation-related dysfunction in AD, but also with our recent observations of the involvement of immune-related transcripts, such as *TYROBP*, *TREM2*, and others [18, 50, 51].

### Validation of modules using gene perturbation signatures

As the co-expression network analysis aims to identify co-regulated modules (or clusters), it is of great interest to evaluate whether the present WCGNA modules indeed capture biological meaningful co-regulation signals rather than random noise. For this purpose, we identified in vitro and in vivo gene perturbation signatures and examined how faithfully the predicted network modules reflected experimental targets in response to the perturbation of each gene.

First, we collected a set of *Tyrobp* gene knock-down (KD) expression signatures derived from mouse microglia cell lines reported in one of our previous studies [18]. This *Tyrobp* KD gene signatures contained 1524 genes which have corresponding human orthologues, among which 1302 human orthologues were covered by the current microarray platforms. In the current dataset, as summarized in Additional file 1: Table S11a, the KD signatures were significantly enriched with *TYROBP*-containing modules by at least 1.7-fold (FDR adjusted *P* value < 0.012) in all 19 brain regions.

Second, we used *PSEN1* mutation gene signatures from a previous study that identified DEGs in familial AD (FAD) caused by *PSEN1* coding mutations [52]. We defined a gene set specific to *PSEN1* mutations by excluding FAD DEGs that were shared with sporadic early onset AD DEG signatures. *PSEN1* gene was represented by four different probesets in the present microarray platforms. Fifty-one modules containing one or more *PSEN1* probesets were identified across 19 brain regions. Forty-five of these modules were significantly enriched for *PSEN1* mutation signatures at FDR adjusted *P* value of 0.05 (Additional file 1: Table S11b).

Third, we re-analyzed the data from a previous study that performed transcriptional profiling of cultured mouse oligodendrocytes with a deletion of the myelination transcription factor *Myrf*, myelin regulatory factor, also known as *C11orf9* [53]. The set of genes differentially expressed in the cells with a *Myrf* deletion compared with the control was significantly enriched in 35 of 45 modules that contained *MYRF* gene across 19 brain regions (Additional file 1: Table S11c).

Overall, we observed strong enrichment of in vivo and in vitro gene perturbation signatures in the network modules harboring the perturbed target. Across different brain regions, the enrichment in target-harboring modules was much stronger than that in modules that did not contain the perturbation target (with one-tailed Wilcox rank sum test *P* value = 3.7 × 10^–10^, 5.8 × 10^–5^, and 7.1 × 10^–16^ for *Tyrobp*, *Myrf*, and *PSEN1*, respectively) (data not shown), suggesting the target genes (i.e. *Tyrobp*, *Myrf*, and *PSEN1*) and their perturbation responders tend to be close in the co-expression network and hence clustered in the same module, thus partly validating the biological meaningfulness of the network modules being tested in the present data. The current validation model is not perfect because a single module cannot capture the whole tissue gene regulatory activity. Thus, we were unable to evaluate the functional role of every module. Nevertheless, the analyses showed that co-expression modules captured network sub-structures of meaningful gene–gene interaction relationships, which was to some degree validated in gene perturbation data for the three genes analyzed here.

### Module relevance to AD pathology and severity of clinical dementia

**Fig. 7:**
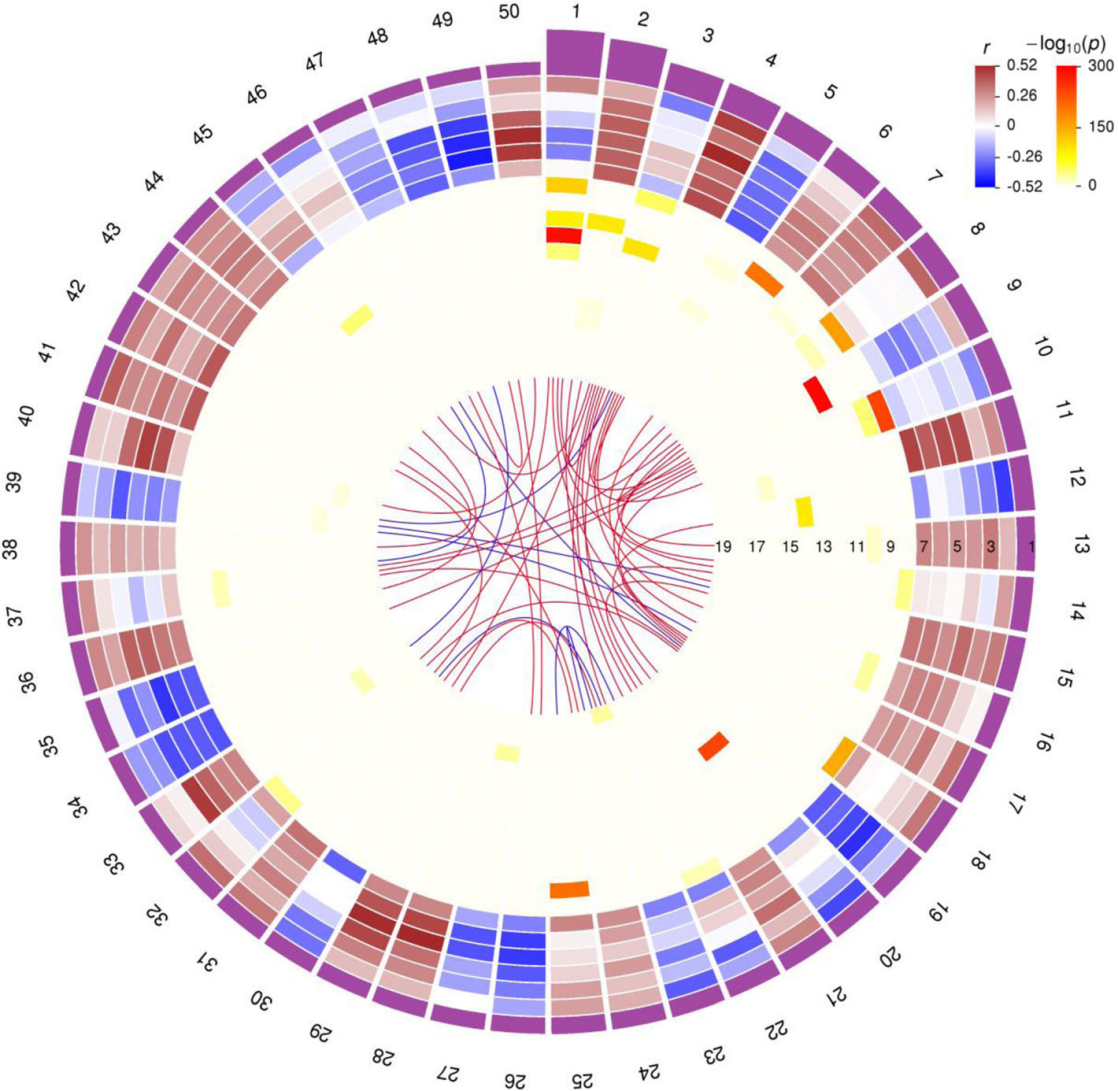
The top 50 ranked modules in the co-expression networks of the 19 brain regions. From outside to inside, the *bar chart* at track 1 shows scaled ranking scores, the *heat maps* at tracks 2–7 show the correlation coefficients (*r*) between module eigengenes and six cognitive/neuropathological traits (in the order of CDR, Braak, CERAD, mean plaque density, sum of NP density estimates, and sum of NFT density estimates), the *heat maps* at tracks 8–13 show − *log*_10_(*P* value) of the enrichment for the DEGs identified for the six traits, the –at tracks 14–19 show − *log*_10_(*P* value) of the enrichment for the genes correlated with the six traits, and the links in the *middle* illustrate the significant correlations (FDR < 0.05; *red* for correlation > 0.8, *blue* for correlation < -0.8) among the modules

The co-expression network structures, and the expression variation underlying the brain networks, collectively reflect molecular processes associated with AD. However, beyond a mere association with AD, the relationship of gene expression perturbations and their modular interrelationships to cognition, cognitive compromise, and the canonical neuropathological lesions of AD is of particular and paramount translational interest. To prioritize the gene modules with respect to their association to AD neuropathology, we ranked the modules by multiple features, including correlations between module eigengenes and cognitive/pathological traits, and enrichment for gene expression signatures such as the DEGs and TCGs calculated above. To measure whether module and phenotypic traits were correlated, we computed the Spearman’s correlation coefficient between module eigengene expression profiles and each trait measure (i.e. dementia severity (CDR), global probability of AD pathology (CERAD), cortical NP density (NP and NPrSum, and neurofibrillary tangle involvement severity (Braak stage and NTrSum). While the modules could be sorted by each individual feature of interest, we performed a comprehensive ranking by aggregating the rankings of all features as described in the “Methods.” The module rankings are provided in Additional file 1: Table S12. Figure 7 shows the ranking of the top 50 modules, with multiple tracks illustrating the different properties of the modules, including ranking score, strength of correlation between eigengene expression and the six traits, significance of enrichment with TCGs and DEGs, and correlations among the module eigengenes.

Table 1 lists the 20 top ranked modules and their brain regions, top functional terms, and module ranking scores. Strikingly, six of the 20 top modules are from one region in the temporal lobe, the inferior temporal gyrus (BM20-ITG), which is consistent with the fact that temporal lobe regions ranked top in relevance to disease as described above. A number of functional pathways known to be implicated in AD were enriched in the top ranked modules. These included: an axonal guidance module (gray17) associated with the superior parietal lobule (BM7-SPL) ranked number 11; a nervous system development module (tan) involving the PT ranked number 12; and a synaptogenesis module (yellow2) in the inferior temporal gyrus (BM20-ITG) ranked number 15. Two cytoskeleton related pathways are present in the top ranked modules, including the number 2 module-tan in BM20-ITG and number 5 module-orchid in the parahippocampal gyrus (BM36-PHG). The relevance of these modules is underscored by a growing body of evidence suggesting that tau accumulates preferentially in axons and may mediate neurotoxicity by altering the organization and dynamics of the actin cytoskeleton and abnormalities of the actin cytoskeleton could be critical in synaptic loss in AD [54, 55]. Two cytoplasm modules were identified in the top 20, including the number 3 module-blue in the inferior frontal gyrus (BM44-IFG) and the number 6 module-salmon in the superior frontal gyrus (BM8-SFG).

**Table 1:** The 20 top ranked modules

Region	Module	Top GO annotation term	Cell type specificity	Risk genes enrich.^a^	Score	Rank
BM44-IFG	Yellow	Nucleus	Astrocytes	IGAP	1	1
BM20-ITG	Tan	Actin cytoskeleton	Oligodendrocytes	Aβ	0.89	2
BM44-IFG	Blue	Cytoplasm	Neurons	IGAP, Aβ	0.62	3
BM20-ITG	Red2	Positive regulation of transcription from RNA polymerase II promoter		-	0.6	4
BM36-PHG	Orchid	Cytoskeleton organization and biogenesis	-	-	0.56	5
BM8-SFG	Salmon	Cytoplasm	-	IGAP, Aβ	0.51	6
BM20-ITG	Mediumblue	Regulation of transcription DNA dependent	-	-	0.5	7
BM7-SPL	Brown	Nucleus	Astrocytes	IGAP	0.5	8
BM32-AC	Purple	Biopolymer metabolic process	-	IGAP	0.46	9
BM8-SFG	Blue	Membrane	Neurons	IGAP, Aβ	0.46	10
BM7-SPL	Gray17	Axon guidance	-	-	0.43	11
PT	Tan	Nervous system development	Neurons	IGAP	0.43	12
BM20-ITG	Maroon	Glycoprotein catabolic process	-	Aβ	0.4	13
BM8-SFG	Green	Nucleus	-	IGAP	0.39	14
BM20-ITG	Yellow2	Synaptogenesis	-	-	0.39	15
BM8-SFG	Pink	Nuclear part	Neurons	Aβ	0.39	16
BM20-ITG	Navy	Positive regulation of cell differentiation	Astrocytes	-	0.38	17
BM17-OVC	Brown	Nucleus	Neurons	IGAP, Aβ	0.38	18
BM4-PCG	Gray24	Regulation of transcription	-	-	0.38	19
PT	Yellow	Macromolecular complex	Neurons	IGAP, Aβ	0.37	20

^**a**^ Indicating whether IGAP or Aβ network genes were enriched in the module

In addition to the pathways implicated in AD previously, we found several novel functional categories enriched in the top modules. The first category, nucleus, was enriched in the top ranked module, yellow in the inferior frontal gyrus (BM44-IFG), and also in the number 8 module (brown) in the superior parietal lobule (BM7-SPL), number 14 module (green) in the superior frontal gyrus (BM8-SFG), and number 18 module (brown) in the occipital visual cortex (BM17-OVC). Nucleus is the organelle of eukaryotic cells in which chromosomes are housed and genes are transcribed. We also identified three transcription regulation modules in the top modules: red2 and mediumblue in the inferior temporal gyrus (BM20-ITG) and gray24 in the precentral gyrus (BM4-PCG). The second category, biopolymer biological process including biopolymer metabolic process, glycoprotein catabolic process and macromolecular complex, was enriched in three modules: the purple module in the anterior cingulate (BM32-AC), maroon module in the inferior temporal gyrus (BM20-ITG), and the yellow module in the PT. Biopolymers are polymeric biomolecules formed in a biological system, such as polypeptides, polynucleotides, and polysaccharides. Essentially, the GO categories biopolymer biological process and cellular component nucleus, which contain 1684 and 1430 genes, respectively, share 706 genes, suggesting that half of the top modules enriched for the two GO categories as well as the transcription regulation pathways likely represented preserved functional networks across different brain regions. As transcriptional and translational dysregulation is expected in AD, some or all members of the biopolymer metabolism/nucleus modules may play important roles in AD pathogenesis.

#### Astrocyte-specific, oligodendrocyte-specific, and neuronal-specific genes are enriched in the top ranked modules

To interrogate the gene expression dataset and determine whether the top ranked modules could be characterized by particular cell types, we overlapped the modules to panels of brain cell type-specific genes (Additional file 1: Table S3). Focusing on the top 20 ranked modules, we found ten modules were enriched for genes expressed in specific cell types at a 5 % FDR (Table 1 and Additional file 1: Table S13). Astrocyte specific genes were enriched in two nucleus modules (yellow in BM44-IFG and brown in BM7-SPL) and one module of positive regulation of cell differentiation (navy in BM20-ITG). Oligodendrocyte specific genes were enriched in the number 2 module actin cytoskeleton (tan) from the inferior temporal gyrus (BM20-ITG). The oligodendroglial myelin-associated pathways were closely linked to the AD-associated neuropathology variables, providing further evidence for targeting oligodendrocyte/myelin disruption as a new therapeutic option to prevent or reverse neuronal impairment leading to AD. Unsurprisingly, neuronal specific genes were enriched in six of the top ranked modules, including a cytoplasm module (blue in BM44-IFG), a membrane module (blue in BM8-SFG), a nervous system development module (tan in PT), a nuclear part module (pink in BM8-SFG), a nucleus module (brown in BM17-OVC), and one macromolecular complex module (yellow in PT). Strong enrichment of astrocyte-specific, oligodendrocyte-specific, and neuron-specific genes in the top ranked modules is consistent with the observation that genes specific to these cell types were enriched for differential expression signatures as shown above. Identification of potential cell types in the top ranked modules associated with AD pathology argues for the development of interventions that target specific molecular pathways in homogeneous cells with increased precision devoid of heterogeneous variation.

#### The top ranked network modules were preserved in an independent (Harvard) brain bank AD dataset

To validate the top ranked networks constructed from the 19 brain regions described herein, we performed in silico analysis of an independent dataset (Fig. 1b). The top ranked modules were first projected onto co-expression networks constructed from the independent Harvard brain bank (HBB) AD dataset [18] to verify whether the top modules were replicable. We assembled the co-expression networks constructed from a combined transcriptome profiling of three brain regions, dorsolateral prefrontal cortex (PFC), visual cortex (OVC), and cerebellum (CB), in 376 late onset AD patients of the HBB AD cohort [18]. For convenience, the network modules identified from the present study sample were referred to as MSBB modules while the network modules from the HBB dataset were referred to as HBB modules. We compared the networks identified from the two datasets and found that 37 % and 25 % of the MSBB modules from BM46-PFC and BM17-OVC, respectively, significantly overlapped with the HBB networks at FDR < 0.05. Conversely, 47 % and 34 % of the HBB modules significantly overlapped with the MSBB BM46-PFC and BM17-OVC networks, respectively. On the other hand, 36 % of all the 1558 MSBB modules significantly overlapped with the HBB networks (Additional file 2: Figure S3) while 77 % of the HBB modules were significantly overlapping with the MSBB networks. If we considered the rankings of networks in relevance to AD pathology in the two datasets, 35.9 % of the top 5 % MSBB modules significantly overlapped with the top 5 % HBB modules, suggesting high consistency of network rankings between independent datasets despite the fact that the HBB data contain only late-stage AD patients while the MSBB data contain the full spectrum of disease status/severity including specimens from normal individuals, persons meeting criteria for mild cognitive impairment and donors in the early stages of AD with only modest AD-associated neuropathology.

Among the top 20 ranked MSBB modules, 13 significantly overlapped with six of the top 20 HBB modules (Additional file 1: Table S14). The most enriched HBB modules were primarily enriched for nerve ensheathment (an oligodendroglial/myelin pathway), cytoskeletal protein binding, cell junction, exocytosis, and oxidoreductase activity, while the corresponding preserved MSBB modules were enriched for actin cytoskeleton, glycoprotein catabolic process, cytoplasm, nucleus, regulation of transcription DNA dependent, nervous system development, macromolecular complex, and biopolymer metabolic process. Taken together, these results highlighted the preserved networks that are promising as targets for the treatment of AD pathology.

#### The top ranked modules were enriched for AD genetic risk factors

The top ranked networks identified above could either play a causal role in AD or be reactive to or independent of the disease. While postmortem gene expression studies cannot directly assess causality of the top ranked networks given by themselves, combinatorial analyses with genetic datasets can help address this question, at least partially. We tested whether the modules identified through analysis of gene expression were enriched for known AD genetic risk factors identified from genome-wide association studies (GWAS). We used a set of AD susceptibility genes from a large scale meta-analysis by the International Genomics of Alzheimer’s Project (IGAP) [56]. We screened for candidate single nucleotide polymorphisms (SNPs) with a nominal *P* value less than 0.05 and then extracted genes near any of the candidate SNPs. We used the relaxed significance threshold for the IGAP gene set with the aim of including de novo causal gene loci of small effect sizes that were unable to exceed the genome wide significance due to insufficient statistical power. A recent study demonstrated that combining genes with nominally significant GWAS *P* values and tissue-specific networks were powerful in building machine learning classifiers for identifying novel genes associated with disease [57]. There was a total of 864 IGAP nominally associated genes that were also profiled in the current microarray gene expression dataset. Table 1 and Additional file 1: Table S15 summarized the enrichment of the IGAP gene sets in the top 20 ranked MSBB modules at an FDR < 5 %. Ten modules from six brain regions were significantly enriched for the IGAP gene set. Four of these modules belonged to the nucleus subnetworks and five were enriched for neuron cell type-specific genes. The top module, yellow from the BM44-IFG network, which was astrocyte cell type-specific and annotated with nucleus function category, was 1.5-fold enriched for IGAP genes (FDR adjusted *P* value = 1.6 × 10^–4^).

One prevalent pathology hypothesis of AD is accumulation of toxic Aβ cascade in the brain. Since its formulation in the early 1990s, the amyloid hypothesis has been somewhat refined but remains the most influential conceptual framework for AD [58]. Centered on Aβ, Campion et al. manually curated a biological network of 335 genes/proteins which have been shown to interfere with Aβ production, clearance, aggregation, or toxicity, including amyloid precursor protein *APP*, beta-secretase *BACE1*, gamma-secretases *PSEN1*/*PSEN2*, and Aβ clearance proteins like *APOE* and *CLU* (reviewed in [59]). Of these 335 genes, 330 were profiled by the current microarray dataset. We assessed whether the 330 Aβ-centered biological network genes were enriched in the top 20 modules and found significant overrepresentation in eight top modules as summarized in Table 1 and Additional file 1: Table S16. Interestingly, five of the eight modules overrepresented with Aβ-centered biological network genes were also enriched for IGAP GWAS gene sets (Table 1), including blue in BM44-IFG, salmon and blue in BM8-SFG, brown in BM17-OVC, and yellow in PT. Note that the *APP* gene was present in one module, blue in BM44-IFG. *APOE* was present in two modules: blue in BM8-SFG and brown in BM17-OVC. Enrichment of the Aβ-centered network in the top modules provided additional support of the relevance of these modules regarding AD pathology.

The strong enrichment of AD genetic risk factors and Aβ-centered network within the top modules further reinforce their strong association with AD. On the other hand, this also identifies other members of these modules that may not only have critical roles in Aβ production and processing, but also represent upstream mechanism that drive Aβ and other pathological processes.

### Selective vulnerability of brain regions in AD

**Fig. 8:**
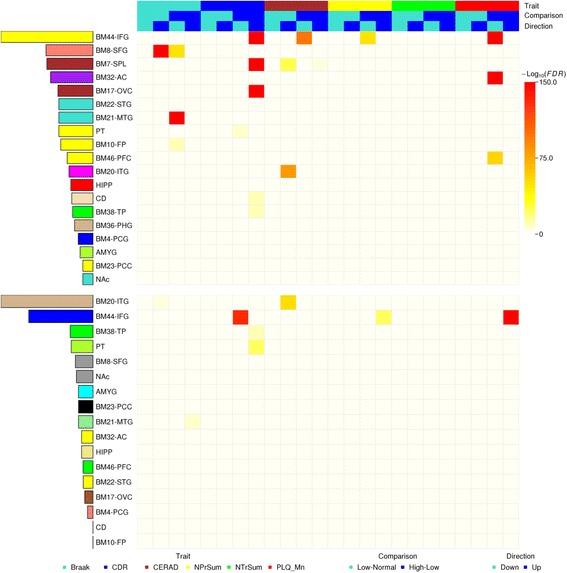
Selective vulnerability of the 19 brain regions to AD as exemplified by actin cytoskeleton (*top panel*) and biopolymer metabolic process (*bottom panel*) subnetworks. The *bar charts* on the *left*, as *colored* by the representative modules’ name, show the region-specific ranking orders. The two *heat maps* illustrate the enrichment of the DEG signatures in the corresponding co-expression modules. The color intensity shows the FDR corrected *P* value of the enrichment test for a module and a DEG signature at minus log10 scale

Functional neuroimaging and neuropathological analyses have shown that different brain regions may have different vulnerability to AD. One of the outstanding questions in studying AD is when (i.e. under what conditions) and where (i.e. which brain regions) the disease emerges. Since this MSBB cohort consists of the subjects from a full spectrum of normal, low, and high severity staging with respect to each cognitive/dementia and neuropathological trait, we explored further the temporal and spatial patterns of the disease by intersecting the gene signatures associated for each trait and the co-expression modules, to relate region-specific subnetworks to the molecular changes at different stage of dementia and neuropathology. As an example, we examined two functional categories across all brain regions: nucleus and actin cytoskeleton, which were enriched in the top two modules and repeatedly observed in the top ranked modules for relevance to AD pathology (Table 1). For each of the 19 brain regions examined, we screened for the MSBB modules significantly enriched for the gene sets related to the two functional categories (FDR < 0.05) and then picked the MSBB module with the highest ranking as the representative of the brain region with respect to the given functional category. We sorted the 19 regions by their region-specific ranking of the representative modules. Meanwhile, we intersected these region-specific representative modules with the previously defined DEG signatures. The results are shown in the bar charts while the intersections between the modules and the DEG signatures are shown in the heat maps in Fig. 8.

Overall, the analysis of region-specific subnetworks showed that different brain regions were affected differently in AD in light of biological processes ranking and enrichment of DEGs. For the nucleus subnetworks, the inferior frontal gyrus (BM44-IFG) was ranked at the top, followed by the superior frontal gyrus (BM8-SFG), the superior parietal lobule (BM7-SPL), and the anterior cingulate (BM32-AC). For the actin cytoskeleton subnetworks, the inferior temporal gyrus (BM20-ITG) was ranked at the top, followed by the inferior frontal gyrus (BM44-IFG), the temporal pole (BM38-TP), and the PT. The subnetworks enriched for the nucleus genes were enriched for differential expression signatures in 16 brain regions. Specifically, the DEGs between the high and low CDR groups were enriched in nine brain region-specific subnetworks, with upregulation or downregulation directions varying among brain regions, suggesting dysregulation of nucleus genes was more likely to be involved in the advanced stage as defined by dementia severity (CDR). For the traits including Braak neurofibrillary pathology stage, CERAD, sum of NP density estimates, and mean plaque density, we found enrichment for the upregulated DEGs between the low and normal groups and the downregulated DEGs between the high and low groups in multiple region-specific subnetworks, suggesting an elevated expression of the nucleus genes in the early stage but reduced expression of the pathway in advanced stage of AD with respect to those traits. Only five region-specific actin cytoskeleton subnetworks showed significant enrichment for DEGs, including the upregulated genes of high–low for Braak neurofibrillary pathology stage, CDR, sum of NP density estimates, and mean plaque density, in regions BM44-IFG, BM21-MTG, BM38-TP, and PT. Specifically, the subnetwork from the top ranked region BM20-ITG was enriched for upregulated DEGs of low–normal as defined by Braak and CERAD, suggesting the upregulation of actin cytoskeleton gene expression might emerge in the early stage (low severity) of disease progression for this particular region. A parsimonious interpretation of these directional changes implicates the region specific pattern of degeneration of cells in the late stages of the disease and the processes that are at play early in the disease before significant degeneration ensues. These opposing directional changes in gene expression based on the early versus late stages of disease highlight the power of this dataset which includes specimens from donors in early stages of disease progression. A focus on the transcriptional changes that accompany the early stages of AD could help identify not only translational treatment targets associated with disease onset, but also suggest the direction of change that treatments should induce to counter disease progression.

## Discussion

This is the first large-scale study to characterize gene expression regulations and also gene transcriptional networks in multiple regions of each neocortical lobe and in subcortical structures in AD. Among the unique features of the present sample cohort is that it contains a continuum spectrum of clinical and neuropathological disease stages from normal to severe. These two features have allowed us to systematically examine the spatial and temporal patterns of molecular pathways and modules in varying physiological states of the disease. We identified more than 6000 probesets which were differentially expressed as a function of cardinal phenotypic features of AD in multiple brain regions (Additional file 1: Table S4a) and also rank-ordered co-expression network modules relevant to AD pathology. By making use of a large-scale human brain single-cell RNA-seq dataset, we identified signatures and network modules with overrepresentation of gene transcripts expressed predominantly in neurons, oligodendrocytes, and astrocytes. To conduct a comprehensive evaluation of the relative involvement of the genes in AD, we ranked the genes by assembling the strengths of association with every trait in every brain region, using a similar ranking metric as that used for network (Additional file 3: Table S17).

Genes are known to be organized into functional networks according to cellular processes and pathways and gene co-expression networks are able to characterize coordinated transcriptional relationships between gene transcripts in various biological contexts including complex diseases [19]. The present study utilizes an integrative network analysis to highlight and prioritize pathways and gene targets underlying AD at different stages of dementia and neuropathology. We rank-ordered the 19 brain regions by systematically comparing the number of gene signatures identified for six phenotypic traits encompassing the cognitive, NFT, and NP dimensions. Interestingly, several top ranked regions (BM36-PHG, BM20-ITG, and BM21-MTG) are located in the temporal lobe including the perirhinal cortex (Fig. 5), a region where tangle pathology is thought to develop early in the disease process. Consistently, half of the top 20 modules were from these same regions, highlighting the significance of these regions in light of disease pathology at the functional pathway level. We verified that the top modules were more likely to be preserved and more than half of them were showing significant overlap with the top ranked modules in an independent AD dataset, underscoring the power of integrative network analysis in revealing functional modules/pathways underlying the disease traits. We identified well established pathways implicated in AD, such as nervous system development, axon guidance, and cytoskeleton, among the top modules expressed in neurons, oligodendrocytes, and astrocytes. In addition, we also identified less studied pathways including biopolymer metabolism and nucleus, providing novel pathway level target to enhance our understanding of the molecular regulation of the disease.

The adult human brain is a complex tissue, comprising multiple cell types with different functions, topologies, and molecular characteristics. As different cell types might present different vulnerability to brain disorder, it is of great value to dissect cell type signals and identify cell type-specific expression change [25, 26, 60]. We utilized a set of cell type-specific genes to identify which cell type-specific marker genes were enriched in the DEGs and the top modules. While tissue homogenate-based postmortem studies preclude definitive resolution of cell type-specific contributions to the disease, the current analysis revealed astrocytes, oligodendrocytes, and neurons specific genes to be enriched for dysregulation. Although the involvement of neurons in the disease process is obvious and expected, a prominent role for the involvement of astrocytes and oligodendrocytes has been postulated less frequently. We also assessed the regional specificity of cell type expression changes. Specifically, the inferior temporal gyrus (BM20-ITG) was enriched primarily for upregulated genes which were overrepresented with oligodendrocyte-specific genes (Additional file 1: Table S6), suggesting that the changes in the nerve ensheathment/oligodendrocyte emerge in this region at an early stage of dementia. Oligodendrocytes coat axons with a fatty sheath of myelin, which promotes faster communication between neurons. Recent evidence suggests that insufficient axon myelination or inability to adequately maintain extant myelin by oligodendrocytes might render the affected axonal processes vulnerable to disease-related damage, such as inflammation, oxidative stress, fibrillogenic Aβ, or to phospho-tau species [61]. Additionally, since oligodendrocytes have been implicated in maintaining axonal integrity, their dysfunction could contribute to neurodegeneration directly. We hypothesize that the disruption of nerve ensheathment/myelin integrity is an early indicator of AD pathology in certain brain regions such as BM20-ITG.

The complex relationship among over 1000 co-expressed gene modules built from 1053 postmortem brain tissues across 19 brain regions was summarized by a meta-co-expression network based on correlations between module eigengene expression profiles. The majority of the meta-modules were brain region specific, i.e. dominated by eigengenes from one brain region, reflecting, on the one hand, strong gene expression correlation within brain regions, and on the other hand, possibly the difference in the biological functions that different brain regions play. However, significant correlations among brain regions were also detected. Specifically, two meta-modules were identified as comprising highly correlated consensus network modules from at least 17 brain regions and these consensus modules were enriched primarily for immune response related functional pathways. This result suggests the immune response is regulated in a coordinated way in different areas of the brain and/or as a result of AD progression throughout the brain. Increasing evidence suggests strong interactions with immunological mechanisms in AD pathogenesis. For example, a number of genes expressed in immune cells of the central nervous system (CNS) carry genetic variants associated with increased risk of AD, including *CD33* [62], *TREM2* [63], and *CR1* [64].

GWAS have been widely employed to identify genetic variants influencing risk for complex diseases, including AD. Although a number of genetic risk loci have been identified, the functional variants and specific genes remain elusive for most loci [65]. Transcriptional profiling enables the capture of a multidimensional view of this complexity, reflecting the interplay of genomic and environmental effects. We examined the expression regulation of 34 AD susceptibility genes and found four genes, *PSEN1*, *MEF2C*, *PICALM*, and *PLD3*, to be differentially expressed in several regions primarily in the advanced stage of disease. While most of the AD susceptibility genes showed no evidence of expression changes, this highlights a big gap between genetic factors and transcription regulations in a complex disease such as AD, further supporting the use of the network analysis that leads to discovery of subnetworks associated with AD clinical and pathological traits.

## Conclusions

In summary, this study provides a comprehensive pan-cortical analysis of genome-wide genes and gene co-expression structures associated with AD pathology in an unprecedented number of brain samples collected from well-characterized individuals with a continuum spectrum of dementia and neuropathology. For the first time we were able to systematically rank-order 44,692 gene probesets, 1558 co-expressed gene modules, and 19 brain regions based upon their association with six AD cognitive and pathological traits. The higher-order network organization of transcriptome uncovered by this study not only narrows down generic pathways to disease-associated specific gene modules but also pinpoints individual genes across 19 brain regions. We validated the network topology of modules using perturbation signatures. More than half of the top ranked gene modules were enriched for AD risk genes and replicated in another independent AD study cohort, further demonstrating the validity and novelty of this study. Such results provide functional contexts for AD risk genes and enable the development of novel hypotheses for further experimental validation. We computed human brain cell type-specific genes from single-cell RNA-seq data and then identified DEG signatures and top network modules specific to neurons, oligodendrocytes, and astrocytes. This study has not only identified novel networks and pathways associated with AD but it has also provided new insights into prominent molecular mechanisms underlying selective regional vulnerability to AD. The data, the results, and the findings from this study have painted a global picture about changes in gene expression and gene–gene interactions in AD and will facilitate future research on the molecular mechanisms of this complex disease and potentially aid in the development of treatment strategies that can target molecular events associated with the earliest documentable stages of disease onset.

## Additional files

Additional file 1: This document contains Supplementary **Tables S1–16**, plus their associated legends/captions. (XLSX 6505 kb)
Additional file 2: This document contains all Supplementary Figures. (DOCX 1540 kb)
Additional file 3: Table S17. Top 10,000 probesets ranked in association with disease traits across 19 brain regions. (XLSX 6710 kb)
