# Supplementary material for: Integrative network analysis of nineteen brain regions identifies molecular signatures and networks underlying selective regional vulnerability to Alzheimer’s disease
Source: Genome Med. 2016 Nov 1;8:104. doi: 10.1186/s13073-016-0355-3 (PMC5088659; doi:10.1186/s13073-016-0355-3)
Supplement: Additional file 2: — This document contains all Supplementary Figures. (DOCX 1540 kb) [file 13073_2016_355_MOESM2_ESM.docx]

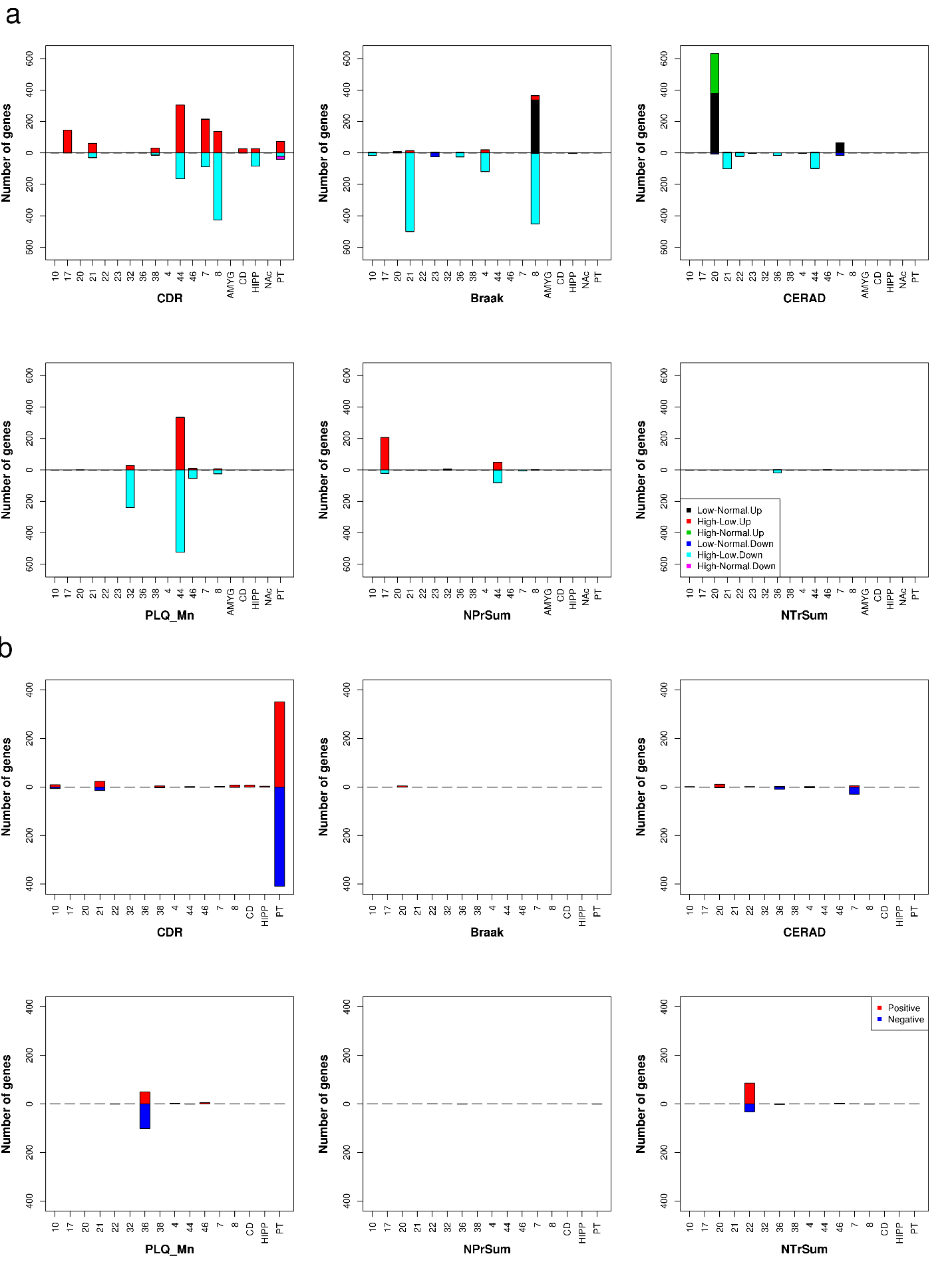


Figure S1 Number of (a) DEGs and (b) TCGs detected in 19 brain regions for 6 traits. The x axis legends denote the brain regions as highlighted in Figure 1a.


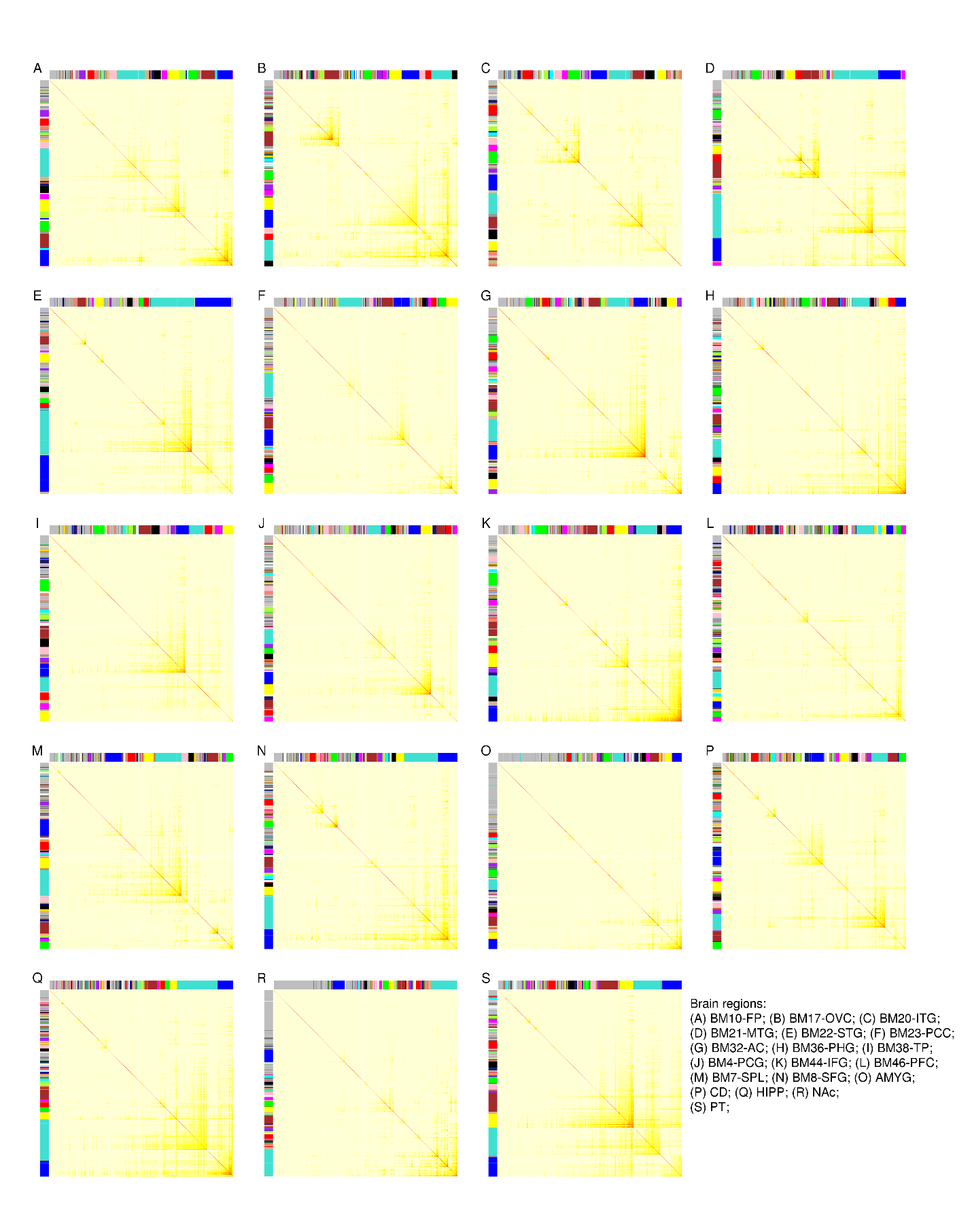


Figure S2 Topological overlap matrix (TOM) plots of the coexpression networks of 19 brain regions. The row and column color labels denote the module membership.


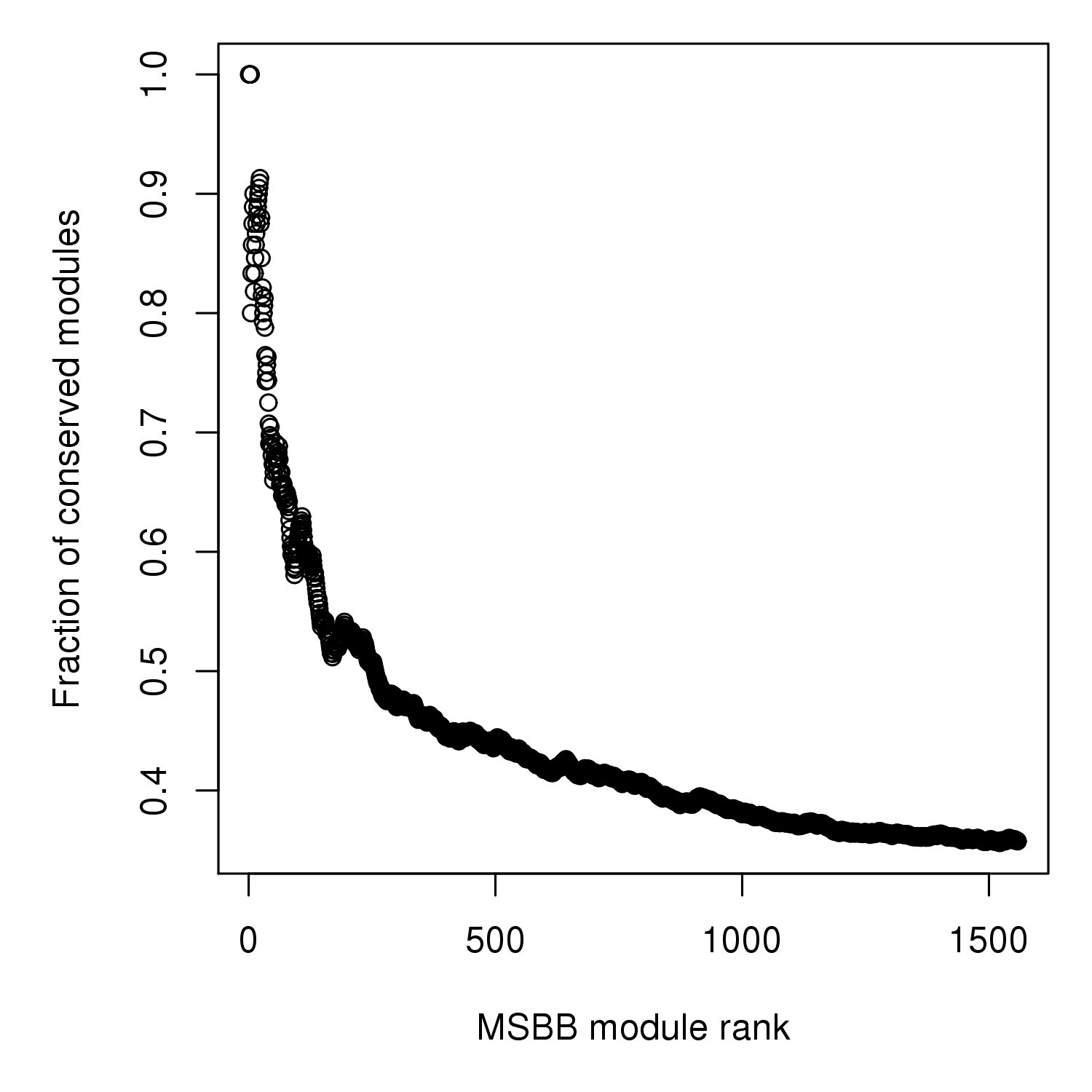


Figure S3 Preservation of MSBB modules in the Harvard brain bank (HBB) AD dataset. The x-axis lists the sorted MSBB modules and each data point denotes the fraction of the MSBB modules up to a given rank that are preserved with one of the HBB modules.
